# Antifungal Activity of the Biphosphinic Cyclopalladate C7a against *Candida albicans* Yeast Forms *In Vitro* and *In Vivo*

**DOI:** 10.3389/fmicb.2017.00771

**Published:** 2017-05-03

**Authors:** Julian E. Muñoz, Diego C. P. Rossi, Kelly Ishida, Cristina C. Spadari, Marcia S. C. Melhem, Daniel M. Garcia, Antonio C. F. Caires, Carlos P. Taborda, Elaine G. Rodrigues

**Affiliations:** ^1^Department of Microbiology, Biomedical Sciences Institute, University of São PauloSão Paulo, Brazil; ^2^Faculty of Health Sciences, Colegio Mayor de Cundinamarca UniversityBogotá, Colombia; ^3^Parasitology Section, Technical Division of Medical Biology, Instituto Adolfo LutzSão Paulo, Brazil; ^4^Department of Pharmacology, Federal University of São PauloSão Paulo, Brazil; ^5^Interdisciplinary Center for Biochemical Investigation, University of Mogi das CruzesMogi das Cruzes, Brazil; ^6^Laboratory of Medical Mycology-LIM53/IMTSP, University of São PauloSão Paulo, Brazil; ^7^Department of Microbiology, Immunology and Parasitology, Paulista School of Medicine, Federal University of São Paulo (EPM-UNIFESP)São Paulo, Brazil

**Keywords:** *Candida* spp., clinical isolates, antifungal chemotherapy, cyclopalladated C7a, vaginal candidiasis, disseminated candidiasis, drug-resistant yeast

## Abstract

Vulvovaginal and invasive candidiasis are frequent conditions in immunosuppressed individuals caused by *Candida albicans* and non-*albicans Candida* spp. Fluconazole and Amphotericin B are the main drugs used to fight the infection. However, resistance to fluconazole and other azole antifungal drugs is an important clinical problem that encourages the search for new therapeutic alternatives. In this work, we evaluate the antifungal activity of the biphosphinic cyclopalladate C7a in the *in vitro* and *in vivo* model. Our results showed fungicidal activity, with low values of minimal inhibitory concentrations and minimum fungicidal concentrations, even for fluconazole and/or miconazole resistant *Candida* isolates. Fluorescence microscopy and transmission electron microscopy revealed that the compound was able to inhibit the formation of hyphae/pseudohyphae and, moreover, promoted morphological alterations in cellular organelles and structures, such as disruption of cell wall, apparent mitochondrial swelling, chromatin marginalization into the nuclei and increased numbers of electron-lucent vacuoles. C7a significantly decreased the biofilm formation and reduced the viability of yeast cells in mature biofilms when tested against a virulent *C. albicans* strain. *In vivo* assays demonstrated a significant decrease of fungal burden in local (vaginal canal) and disseminated (kidneys) infection. In addition, we observed a significant increase in the survival of the systemically infected animals treated with C7a. Our results suggest C7a as a novel therapeutic agent for vaginal and disseminated candidiasis, and an alternative for conventional drug-resistant *Candida*.

## Introduction

Invasive candidiasis is a disease caused by the fungal genus *Candida*. It is a serious infection that can affect the blood, heart, brain, eyes, bones, and other tissues (Antinori et al., 2016). Moreover, it is the most common fungal disease among hospitalized, oncologic and immunosuppressed patients in the developed world (Antinori et al., 2016). The mortality among patients with invasive candidiasis is as high as 40%, even when patients receive treatment (Hassan et al., 2009). Mucosal infections are also very common, with vulvovaginal candidiasis (VVC) being the second most frequent gynecological condition after bacterial vaginosis (Anderson et al., 2004). VVC is a disease that significantly impacts the quality of life of affected women worldwide, particularly those with chronic and recurrent infections (Sobel, 2016).

Common therapies for invasive candidiasis and mucocutaneous infections, such as VVC, include treatment with polyenes (nystatin, amphotericin B and its lipid formulations), azoles (fluconazole and voriconazole) and echinocandins (caspofungin) (Pappas et al., 2016). Although the efficacy of currently used anticandidal drugs is satisfactory, there are reports of infections by multidrug-resistant *Candida albicans* (Pappas et al., 2016). Moreover, these antifungals have various drawbacks regarding their spectrum of activity, pharmacokinetic properties and host toxicity, suggesting that the discovery of new antifungal compounds is urgently needed (Campoy and Adrio, 2016).

Cancer chemotherapeutic agents based on transition metals, especially those of the platinum group, are effective against tumors, however, come with the cost of an extremely high toxicity to the patient (Harper et al., 2008). In the search for alternative drugs containing a transition metal of the same group but with reduced toxicity and a broader spectrum of activity for cancer treatment, palladium (II) complexes were found to be promising candidates *in vitro*. Due to their extremely high lability under physiological conditions, cyclopalladated compounds complexed with a biphosphinic group were synthetized. Given the increased stability of the complexes, the effective dose was able to be lowered, thereby significantly reducing the toxicity of the drug (reviewed in Caires, 2007). One of these complexes, the biphosphinic cyclopalladated C7a, {Pd_2_ [*S*_(-)_C^2^, N-dmpa]_2_ (μ-dppe)Cl_2_}, showed strong activity *in vitro* and *in vivo* against murine melanoma, several human tumor cells (Rodrigues et al., 2003; Serrano et al., 2011) and in a patient-derived xenograft model of Adult T Cell Leukemia/Lymphoma (Guimaraes-Correa et al., 2011). Interestingly, low doses of C7a were also effective *in vitro* and *in vivo* against *Trypanosoma cruzi* (Matsuo et al., 2010) and the fungi *Paracoccidioides brasiliensis* and *Paracoccidioides lutzii* (Arruda et al., 2015). *In vitro*, C7a reduced colony forming units (CFU) of *Cryptococcus neoformans* and *Candida albicans* (Arruda et al., 2015).

In tumor cells, C7a induced apoptosis by dissipating the mitochondrial membrane potential, thereby activating effector caspases, causing chromatin condensation and DNA degradation (Rodrigues et al., 2003; Guimaraes-Correa et al., 2011; Serrano et al., 2011). In *Trypanosoma cruzi*, the compound evokes an apoptosis-like cell death through mitochondrial disruption (Matsuo et al., 2010). Likewise, in *Paracoccidioides lutzii* and *Paracoccidioides brasiliensis* isolates, C7a promotes apoptosis and autophagy-like cell death *in vitro* (Arruda et al., 2015).

In this study, we demonstrate that C7a is effective *in vitro* against several strains of *Candida*, including azoles resistant strains. Besides inhibiting the pseudohyphae/hyphae formation by *Candida* yeast cells, the compound induced morphological alterations suggestive of cell death. C7a also decreased the biofilm formation by *Candida albicans* and reduced the viability of yeast cells forming mature biofilms. Furthermore, C7a proved to be effective at controlling vaginal and systemic *Candida* infections in experimental models. It is therefore a novel and promising candidate for candidiasis treatment and an alternative compound for use in the treatment of the multi-drug resistant isolates.

## Materials and Methods

### Microorganisms

The clinical isolates of fluconazole-susceptible and resistant *Candida* were obtained from Instituto Adolfo Lutz (São Paulo, Brazil): *Candida albicans* (*n* = 14), *Candida parapsilosis* (*n* = 9), *Candida tropicalis* (*n* = 10), *Candida glabrata* (*n* = 10), and *Candida krusei* (*n* = 10). Standard strains of *C. albicans* (SC5314, ATCC 10231 and ATCC 24433), *C. parapsilosis* (ATCC 22019), *C. tropicalis* (ATCC 200956 and ATCC 28707), *C. glabrata* (ATCC 2001) and *C. krusei* (ATCC 6258) were also included. All strains were maintained at -80°C. To perform the experiments, the fungi were subcultured in Sabouraud dextrose broth (Becton, Dickinson and Company; Sparks, NV, USA) at 37°C for 24 h at 150 rpm before each assay.

### Cyclopalladated Compound

The biphosphinic cyclopalladated complex C7a was synthesized from N,N-dimethyl-1-phenethylamide (dmpa) and complexed to 1,2 ethanebis (diphenylphosphine, dppe) ligand, as previously described (Rodrigues et al., 2003). The compound was diluted to a final concentration of 10 mM in DMSO (cell culture tested, Sigma–Aldrich, St. Louis, MO, USA), and for *in vitro* and *in vivo* assays, diluted to the final concentration in culture media or PBS, respectively. For use in the vaginal candidiasis model, C7a (2.5 and 5% w/w) was incorporated into a vaginal cream (10% wax self-nonionic emulsifier, 2% mineral oil, 5% propylene glycol and 84% distilled water, pH 4.5) and applied topically (Rossi et al., 2012).

### Standard Antifungal Agents

Three antifungal agents were used as references: amphotericin B (AMB; Sigma–Aldrich) diluted in DMSO to obtain the concentration of 1,600 μg/mL; miconazole (MCZ; Sigma–Aldrich) diluted in DMSO to obtain the concentration of 1,600 μg/mL; and fluconazole (FCZ; Pfizer, São Paulo, Brazil) diluted in sterile water to obtain the concentration of 2,560 μg/mL. The stock solutions were stored at -20°C. For *in vitro* and *in vivo* assays, antifungals were diluted to the final concentration in culture media or PBS, respectively. For use in the vaginal candidiasis model, MCZ (2% w/w) was incorporated into the vaginal cream described above.

### Antifungal Susceptibility Assay on Planktonic Cells of *Candida*

The antifungal susceptibility of planktonic cells of *Candida* spp. clinical isolates and standard strains was assessed by broth microdilution assay as described in the protocols M27-A3 (Clinical, and Laboratory Standards Institute [CLSI], 2008) and M27-S4 (Clinical, and Laboratory Standards Institute [CLSI], 2012), according to the Clinical and Laboratory Standards Institute.

The IC_50_ and IC_90_ values were determined by spectrophotometric reading at 492 nm in a microtiter plate reader (Epoch, Biotek Instruments, USA). The minimum inhibitory concentration (MIC, Supplementary Table S1) was defined as the lowest concentration inhibiting 90% (IC_90_, for polyene agents) and 50% (IC_50_, for azole agents) of fungal growth (Clinical, and Laboratory Standards Institute [CLSI], 2008). For C7a compound, MIC values were defined as the IC_90_. As fungal populations were higher than 10 strains, MIC_50_ and MIC_90_ could be determined and are defined as the concentrations that inhibit 50 and 90% of population, respectively (**Table 1**).

**Table 1:** Values of minimum inhibitory concentration (MIC, expressed in μg/ml) of the C7a, AMB and MCZ and FCZ determined in clinical isolates of *Candida* spp.

Strains	Antifungal drugs	Concentrations (μg/mL)		
		
		MIC Range	MIC_50_	MIC_90_
All species	**C7a**	0.25–4	1	4
	**AMB**	0.125–16	1	1
	**MCZ**	0.03–>16	0.25	4
	**FCZ**	0.03–>64	1	>64
*Candida albicans*	**C7a**	0.25–1	0.5	1
	**AMB**	0.5–1	1	1
	**MCZ**	0.03–4	0.03	0.5
	**FCZ**	0.03–>64	0.25	64
*Candida tropicalis*	**C7a**	0.5–2	1	2
	**AMB**	0.5–2	1	2
	**MCZ**	0.06–8	0.5	8
	**FCZ**	0.25–>64	1	64
*Candida parapsilosis*	**C7a**	1–4	2	4
	**AMB**	0.5–1	1	1
	**MCZ**	0.06–4	0.25	4
	**FCZ**	0.06–>64	1	16
*Candida glabrata*	**C7a**	0.25–4	1	2
	**AMB**	0.125–2	0.5	1
	**MCZ**	0.03–16	0.03	0.5
	**FCZ**	0.25–>64	2	8
*Candida krusei*	**C7a**	1	1	1
	**AMB**	0.5–1	0.5	1
	**MCZ**	2–8	4	8
	**FCZ**	64–>64	64	>64


****All species*,** represents the combination of all clinical isolates in this study.*

To evaluate the minimum fungicidal concentration (MFC), aliquots (10 μL) were taken from each well of the broth microdilution assay, plated in drug-free media (Sabouraud dextrose agar) and incubated at 37°C for 48 h. The MFC was defined as the lowest concentration that completely inhibited the formation of colonies. The cumulative percentage of isolates inhibited by C7a or AMB, the most effective compounds, for each dilution is presented in **Table 2**. For MIC and MFC determinations, each point was performed in triplicate. MIC and MFC results shown in Supplementary Table S1 represent the median value of 3 experiments executed.

**Table 2:** Cumulative percentage of the minimum fungicidal concentration (MFC) of C7a and AMB determined in clinical isolates of *Candida* spp.

		Cumulative Percentage of MFC (%)								
		
MFC (μg/mL)		0.12	0.25	0.5	1	2	4	8	16	>16
All species	**C7a**		11.5	31.2	72.2	78.7	86.9	90.2	93.5	100
	**AMB**			13.1	67.1	83.5	86.8	86.8	86.8	100
*C. albicans*	**C7a**		41.2	88.1	100					
	**AMB**			11.7	58.7	64.5	64.5	64.5	64.5	100
*C. parapsilosis*	**C7a**				10	10	50	70	90	100
	**AMB**			10	40	90	100			
*C. tropicalis*	**C7a**			16.6	66.6	91.6	91.6	91.6	91.6	100
	**AMB**				83.3	83.3	83.3	83.3	83.3	100
*C. glabrata*	**C7a**			18.1	81.7	81.7	90.7	90.7	90.7	100
	**AMB**			9	72.6	90.7	100			
*C. krusei*	**C7a**			0	81.8	90.8	90.8	90.8	90.8	100
	**AMB**			36.3	81.7	100				


*Results represent the percentage of isolates showing total growth inhibition at each compound concentration.***All species*,** represents the combination of all clinical isolates in this study.*

### Fluorescence Microscopy

*Candida albicans* ATCC 10231 yeasts (10^3^ CFU/mL) were treated with 0.5 μg/mL of C7a for 24 h at 37°C in RPMI 1640 media buffered with MOPS 0.16 M. After this period, C7a-treated and untreated cells were washed with PBS pH 7.2, fixed with 4% paraformaldehyde in PBS for 30 min, adhered to glass coverslips previously covered with poly-L-lysine and stained with Calcofluor-White 1 mg/mL (Sigma–Aldrich) for 5 min. The coverslips were washed and mounted in n-propyl gallate solution (1%) and observed under EVOS FL microscope (AMG, WA, EUA). For quantification, about 300 cells were counted in random fields in the untreated and C7a-treated groups. Result shows representative images of two independent experiments.

### Transmission Electron Microscopy

*Candida albicans* ATCC 10231 treated 24 h with 0.5 μg/mL C7a were fixed with 2.5% glutaraldehyde in 0.1 M cacodylate buffer (pH 7.2) for 2 h at room temperature. Post-fixation was carried out in 1% osmium tetroxide in cacodylate buffer containing 1.25% potassium ferrocyanide and 5 mM CaCl_2_ for 2 h. Thereafter, the cells were dehydrated with increasing ethanol concentrations, 100% propylene and embedded in Spurr’s resin. Ultrathin sections were stained with uranyl acetate and lead citrate, and observed in a transmission electron microscope (model Jeol 100CX, JEOL, Japan).

### Biofilm Minimal Inhibitory Concentration (BMICs)

The minimum concentration of C7a and AMB that inhibited the biofilm formation and preformed biofilm cell viability was verified as previously described (Pierce et al., 2008). To evaluate the effect of C7a and AMB in preventing the biofilm formation, 50 μL of each compound serially diluted in RPMI 1640 media (Sigma–Aldrich) buffered with 0.16 M MOPS pH 7.0, were added to plates containing 50 μL of *C. albicans* ATCC 10231 2 × 10^6^ CFU/mL in a 96-well plate. To evaluate the efficacy of the compounds against preformed biofilms, biofilms grown for 24 h were gently washed and 100 μL of serially diluted compounds was added. After the addition of C7a or AMB, the plates were sealed with parafilm and incubated at 37°C for 24 h. Plates were washed twice with PBS to remove non-adherent cells and the XTT viability test was performed to determine the metabolic activity of the biofilm. The positive control was the viability of untreated yeast cells and the negative control was the absorbance of the medium alone. BMIC was defined as the first concentration of C7a that reduced the metabolism of yeast cells in the biofilm compared to the positive control. Results show one representative experiment out of three independent experiments executed, samples in quadruplicates.

### *In Vivo* Infections and Treatments

Isogenic BALB/c mice (6–8 weeks old females) were bred at the University of São Paulo, Brazil, in an animal facility under specific pathogen-free conditions. The procedures involving animals and their care were conducted according to the local ethics committee and international rules and all experiments were approved by the Institutional Animal Care and Use Committee of Institute of Biomedical Sciences (ICB), University of São Paulo (USP).

Disseminated candidiasis in immunocompetent animals was induced by intravenous inoculation (tail vein) of 3 × 10^6^ *C. albicans* ATCC 10231 yeast cells suspended in 100 μL of PBS on day 0. Mice were treated intraperitoneally (i.p.) with PBS (control), C7a (0.3 mg/kg) or FCZ (20 mg/kg) every day for 7 days, starting on day 1. Animals were sacrificed 8 days after infection and kidneys were collected for fungal burden determination. Results show one representative experiment out of three independent experiments executed.

For disseminated candidiasis in immunosuppressed animals, two doses of 100 mg/kg cyclophosphamide (Sigma–Aldrich) were administered i.p. 4 days and 1 day before infection with *C. albicans*, then again on day 3 post infection and every 4 days thereafter (Rossi et al., 2012). The animals were kept in cages lined with wood shavings and closed with an autoclaved filter, and served autoclaved food and water in order to maintain a sterile environment. Cages were exchanged twice a week in laminar flow hoods. The animals were considered anergic when the number of blood leukocytes was found to be fewer than 100 cells/mm^3^ (Andes et al., 2008). On day 0, animals were intravenously (i.v.) infected with 1 × 10^4^ *C. albicans* ATCC 10231 yeast cells suspended in 100 μL of PBS. Mice were treated i.p. with PBS (control), C7a (0.3 mg/kg) or FCZ (20 mg/kg), every day for 7 days, starting on day 1. The survival of the animals was evaluated for 30 days after infection. Results show one representative experiment out of three independent experiments executed.

For vaginal candidiasis, the pseudo-oestrus phase was induced by subcutaneous administration of 0.5 mg of 17 beta-estradiol valerate (Sigma–Aldrich) dissolved in sesame oil (Sigma–Aldrich) 3 days before vaginal infection (Hamad et al., 2004). Mice were infected intravaginally with 3 × 10^6^ *C. albicans* ATCC 10231 yeast cells suspended in 10 μL of PBS, on day 0. Topical treatment was performed with C7a and AMB incorporated in a vaginal cream, as described above, every day for 7 days, starting on day 1. The vaginas were collected on day 8 for fungal burden determination and histopathological analysis. Results show one representative experiment out of three independent experiments executed.

### Fungal Burden in Organs of Infected Mice

Mice were sacrificed 8 days after intravenous or vaginal infection and kidneys and vaginas, respectively, were removed and weighed. Tissues were individually homogenized by mechanical disruption in 1 mL of PBS and 100 μL of these suspensions were inoculated in plates containing brain-heart infusion (BHI) agar media. Colonies were counted visually after 24 h of incubation at 37°C.

### Histopathology

The vaginal canal was excised 8 days after infection, fixed in 10% buffered formalin, and embedded in paraffin for sectioning. Sections were stained with Periodic acid–Schiff (PAS) and examined microscopically at 100× magnification (Optiphot-2, Nikon, Tokyo, Japan).

### Statistical Analysis

Statistical analyses were performed using GraphPad Prism version 6.0 (GraphPad Software, San Diego, CA, USA). Statistical comparisons were made by analysis of variance (one-way ANOVA) followed by a Tukey-Kramer post-test. *P*-values of <0.05 indicated statistical significance. Survival curves were analyzed by the Log-rank (Mantel–Cox) test and *p*-values of ≤0.05 were used to indicate statistical significance. A 95% confidence interval was determined in all experiments.

## Results

### Anticandidal Activity of Compound C7a *In Vitro*

Susceptibility of the clinical isolates and ATCC standard strains of *C. albicans*, *C. tropicalis*, *C. parapsilosis*, *C. glabrata*, and *C. krusei* to C7a *in vitro* is compiled in Supplementary Table S1 and **Tables 1**, **2**.

Supplementary Table S1 shows the MIC and MFC values for each isolate individually. Amongst clinical isolates and ATCC standard strains of all *Candida* species there are two multidrug-resistant strains (*C. tropicalis* ATCC 200956 and ATCC 28707) and one resistant isolate only to AMB (*C. glabrata* IAL-23); however, there are several isolates resistant to FCZ, including all *C. krusei* isolates (Supplementary Table S1).

Also of note is that FCZ- and MCZ-susceptible isolates (low MIC values) showed elevated MFC values for at least one of these drugs (>16 or >64), suggesting a fungistatic effect (Supplementary Table S1). Most of *Candida* strains were susceptible to low concentrations of C7a as well as AMB (MFC values are close to MIC values) suggesting a fungicidal action (Supplementary Table S1).

When all individual values of clinical isolates are grouped (*All species*, **Table 1**) and MIC values are evaluated, it is of notice that C7a and AMB showed a short MIC range, 0.25–4 μg/mL and 0.125–16 μg/mL, respectively. MCZ and FCZ showed a wider range of MIC values, 0.03–>16 μg/mL and 0.03->64 μg/mL, respectively, suggesting that the susceptibility/resistance response of clinical isolates to these azole compounds are highly variable (*All species*, **Table 1**). Individual *Candida* species showed similar results (**Table 1**). The fungicidal effect of the most active compounds, C7a and AMB, is shown as the cumulative percentage of MFC in **Table 2**. Considering grouped values of clinical isolates (*all species*, **Table 2**), C7a was more efficacious than AMB at low concentrations (0.25 and 0.5 μg/mL), however, at higher concentrations both compounds demonstrated similar effect killing most of isolates (≥80%). It is important to note that C7a was more efficacious against *C. albicans* than AMB: at concentrations of 0.25 and 1 μg/mL C7a killed 41.2 and 100% of isolates, respectively, whereas AMB at the concentration of 0.5 μg/mL eliminated only 11.7% of isolates and 100% of isolates were killed only in concentrations higher than 16 μg/mL (**Table 2**). However, AMB was more efficacious against isolates of *C. parapsilosis* (**Table 2**). For the other *Candida* species, the two compounds showed similar fungicidal efficacy (**Table 2**). When MFC values of compounds were compared, there was no significant difference between C7a and AMB (*P* = 0.8399, Supplementary Table S1). Significant differences were found when C7a was compared to FCZ or MCZ (*P* < 0.0001, Supplementary Table S1).

### Morphological Alterations of *C. albicans* Treated with C7a

**FIGURE 1:**
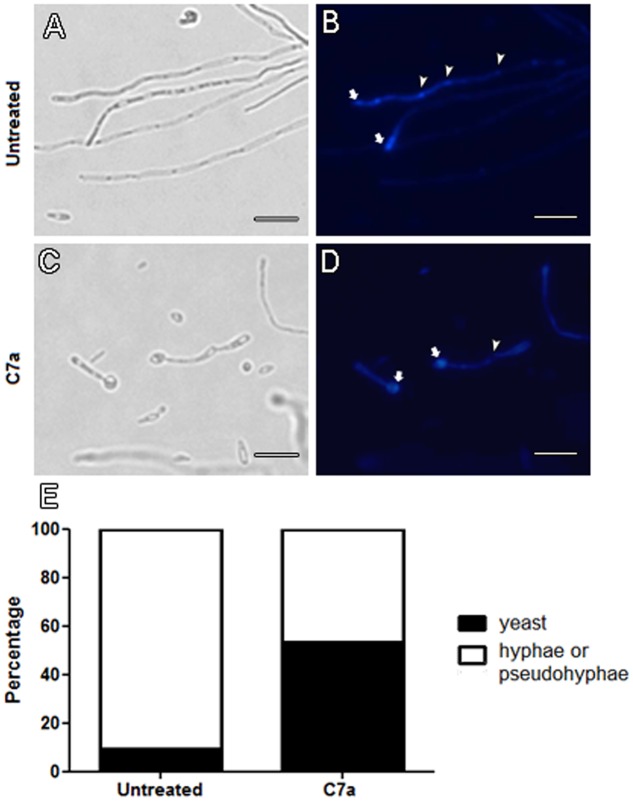
**Hyphae/pseudohyphae formation is impaired in C7a-treated cells.** Yeast cells of *Candida albicans* ATCC 10231 were treated with 0.5 μg/mL of C7a for 24 h at 37°C in RPMI 1640 buffered with MOPS 0.16 M. Thereafter, untreated cells **(A,B)** and cells treated with C7a **(C,D)** were stained with Calcofluor White. Arrows in **(B,D)** show Calcofluor White staining at the end of hyphae and yeast-mother cell, respectively. Arrowheads in **(B)** show chitin-rich septa stained by Calcofluor White and in **(D)** absence of this structure. Yeasts and hyphae/psedohyphae were quantified as described in section “Material and Methods” **(E)**. Bars = 20 micrometers.

**FIGURE 2:**
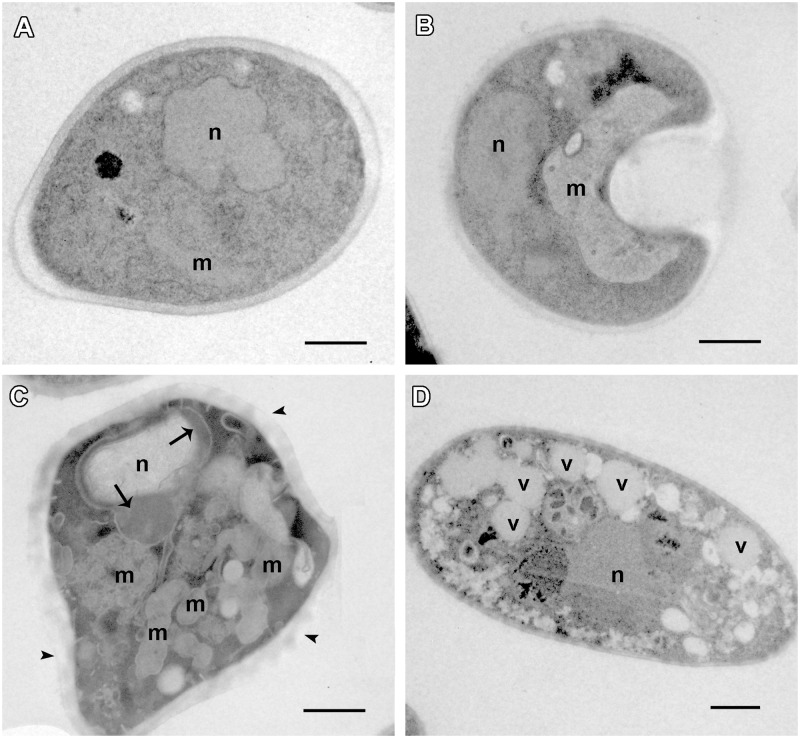
**Morphological alterations were observed in C7a-treated cells.** Transmission electron microscopy of *Candida albicans* ATCC 10231 yeast cells untreated **(A)** and treated with 0.5 μg/ml of C7a **(B–D)** for 24 h at 37°C in RPMI 1640 buffered with MOPS 0.16 M. n, nucleus; m, mitochondria; v, vacuole. Bars = 1 micrometer. DNA marginalization is pointed with arrows and damage at cell wall with arrowheads in **(C)**.

Treatment of *C. albicans* with 0.5 μg/mL C7a induced morphological modifications on yeast cells *in vitro*. Fluorescence microscopy with Calcofluor White of C7a-treated cells revealed that the compound was able to decrease (40%) the formation of hyphae/pseudohyphae compared to untreated cells (**Figure 1E**). This is supported by the observation of an intense staining of Calcofluor White in the yeast-mother cell in treated cultures (arrows, **Figure 1D**), while staining of untreated cells occurred at the end of hyphae corresponding to the cell apical growth region (arrows, **Figure 1B**). In addition, septa are formed in the untreated cells in contrast to the absence of septa in the C7a-treated cells (arrowheads in the **Figures 2B,D**, respectively). These results suggest that C7a affects the formation of hyphae/pseudohyphae by yeast cells in culture.

The morphological effects induced in *C. albicans* by the treatment with C7a were also evaluated using transmission electron microscopy which showed important alterations in some organelles and structures that may be potential targets for C7a. Untreated cells of *Candida albicans* ATCC 10231 presented a normal cell ultrastructure, such as compact and electron-dense cell wall, cytoplasmic membrane, homogeneous and electron-dense cytoplasm, nucleus and mitochondria (**Figure 2A**). Cells treated with 0.5 μg/mL of C7a for 24 h at 37°C exhibited a damaged cell wall (arrowheads in the **Figure 2C**). In addition, increased numbers of electron-lucent vacuoles (**Figure 2D**), DNA alterations as marginalization of genetic material in the nucleus (arrow in the **Figure 2C**) and apparent mitochondrial swelling were observed in C7a-treated cells (**Figures 2B,C**). Additional images are shown in **Supplementary Figure S1**.

### Effect of C7a on *C. albicans* Biofilm Formation and Mature Biofilms *In Vitro*

**FIGURE 3:**
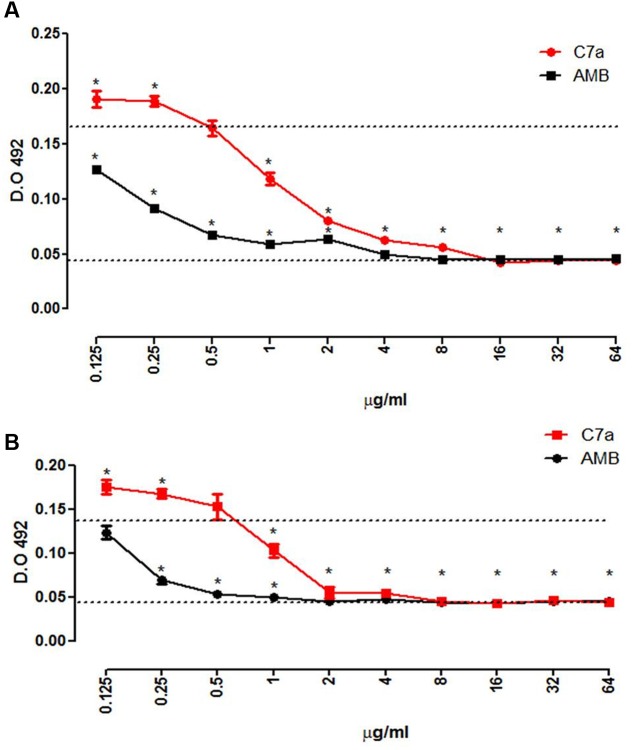
**C7a affects the maturation process and the mature biofilm.** Effect of C7a or AMB in the mature biofilm **(A)** and in the maturation of the biofilm **(B)** of *C. albicans* ATCC 10231 was determined by XTT reduction assay. Upper dotted line represents the untreated positive control, and the lower dotted line represents the negative control. Mean and standard deviation of each point is shown. ^∗^*P* < 0.05, determined by analysis of *t*-test two-tailed compared to the positive control.

The BMIC, minimum concentration of C7a that significantly reduced the metabolism of yeasts in a mature biofilm (**Figure 3A**) and reduced the biofilm formation (**Figure 3B**), was 1 μg/mL. Although effective, C7a was less effective than AMB which interfered with the mature biofilm and biofilm formation of *C. albicans* ATCC 10231 yeast cells in concentrations 8 and 4 times lower, respectively.

### Evaluation of the Antifungal Activity of C7a *In Vivo*

**FIGURE 4:**
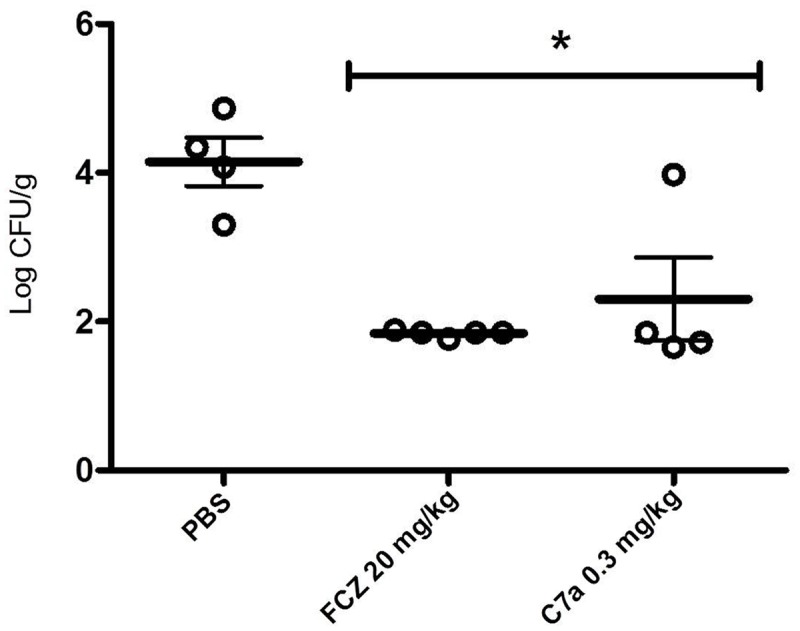
**Cyclopalladated C7a treatment of disseminated candidiasis.** The disseminated candidiasis was induced by intravenous injection of 3 × 10^6^ *C. albicans* ATCC 10231 yeasts in female BALB/c mice. Intraperitoneal treatment was carried out every 24 h for 7 days after infection, and animals were inoculated with PBS, 0.3 mg/kg of C7a, or 20 mg/kg of FCZ. Treatment was evaluated by determination of the number of colony forming units (CFU) per gram of tissue of the kidneys, 8 days after infection. Animals are represented individually, and 1 representative experiment out of 3 is showed. ^∗^Indicates statistical significance (ANOVA with post-Tukey test, *P* < 0.05) compared to PBS-inoculated group.

Previously immunosuppressed female BALB/c mice were injected in the tail vein with 1 × 10^4^ *Candida albicans* ATCC 10231 yeast cells, and were treated i.p. with 0.3 mg/kg of C7a or 20 mg/kg of FCZ every 24 h for 7 days. On day 8, the fungal load of treated and untreated animals was evaluated by quantifying the CFUs from recovered kidneys. The treatment with C7a or FCZ showed a significant reduction (*P* < 0.05) in the fungal burden when compared with untreated mice (**Figure 4**). Although a dose 66 folder high of FCZ was used (Rossi et al., 2012) the fungal burden reduction induced by both compounds was similar (**Figure 4**).

**FIGURE 5:**
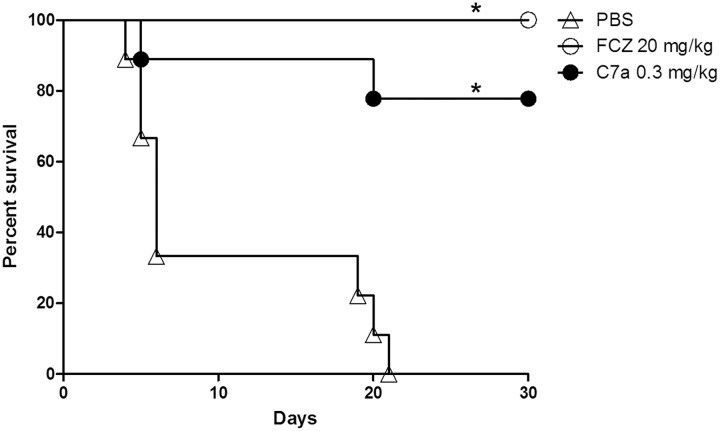
**Survival of immunosuppressed mice with disseminated candidiasis treated with C7a or FCZ.** Previously immunosuppressed animals were intravenously infected with 10^4^ yeasts of *C. albicans* ATCC 10231. The animals (*n* = 10/group) were intraperitoneally treated with PBS, 0.3 mg/kg of C7a or 20 mg/kg of FCZ every 24 h for 7 days. Animal survival was observed for 30 days after infection. ^∗^Indicates statistical significance compared to PBS-inoculated group (Long-rank test, *P* < 0.05). One representative experiment out of 3 is showed.

The effect of C7a treatment on survival of immunosuppressed animals infected systemically with *Candida albicans* was assessed. The group of infected animals treated with PBS reached 100% mortality 21 days post infection. Treatment of infected animals with 0.3 mg/kg of C7a produced a survival rate of 78% and treatment with 20 mg/kg of FCZ produced a survival rate of 100% within 30 days post infection (**Figure 5**).

**FIGURE 6:**
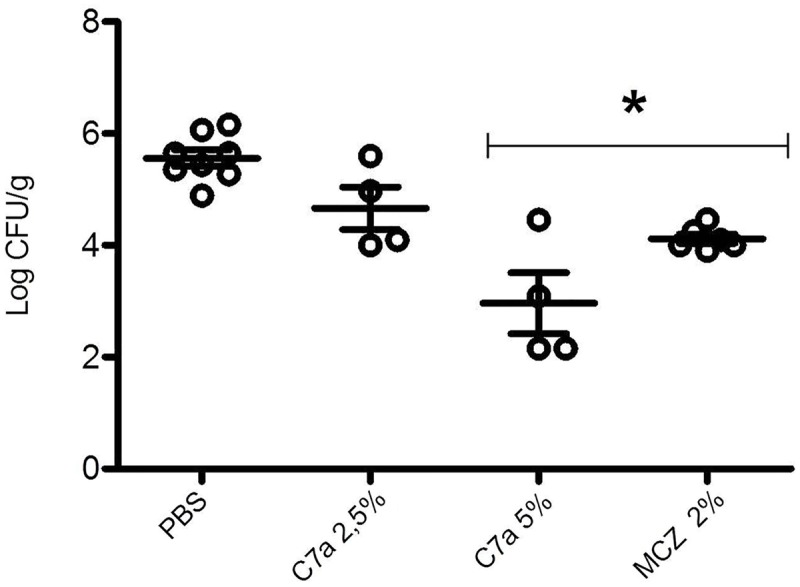
**Cyclopalladated C7a treatment of vaginal candidiasis.** The vaginal candidiasis was induced by local inoculation of 3 × 10^6^ *C. albicans* ATCC 10231 yeasts suspended in 10 μL of PBS in BALB/c mice. Animals were treated every 24 h for 7 days with a vaginal cream containing PBS, 2.5% or 5% of C7a, or 2% of MCZ. Treatment was evaluated by determination of the number of colony forming units (CFU) per gram of tissue of the vaginas, 8 days after infection. Animals are represented individually, and 1 representative experiment out of 3 is showed. ^∗^Indicates statistical significance (ANOVA with post-Tukey test, *P* < 0.05) compared to PBS-inoculated group.

**FIGURE 7:**
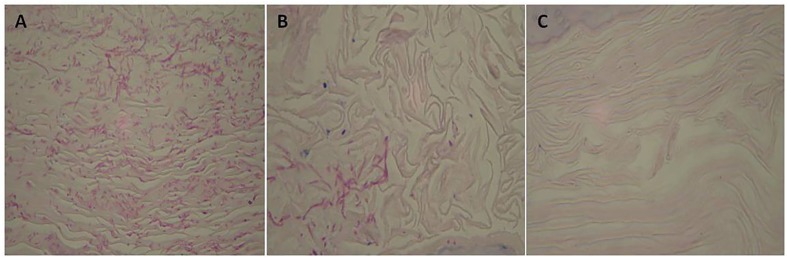
**Histological sections of vaginal canal from BALB/c female mice infected intravaginally with 3 × 10^5^*C. albicans* ATCC 10231 yeast cells and treated with a topical cream containing MCZ (2%) or C7a (5%).** Animals were treated every 24 h for 7 days, vaginal canals were removed after 8 days of infection. **(A)** control group, infected animals and treated with vehicle alone, **(B)** mice infected and treated with MCZ, **(C)** mice infected and treated with C7a. PAS staining of the tissue sections and 100× magnification. Representative images of 10 animals per group.

Treatment of vaginal candidiasis with a topical cream containing 5% (but not 2.5%) of C7a or 2% of MCZ led to a significant (*P* < 0.05) decrease in vaginal CFU when compared to untreated mice (**Figure 6**). Histopathological analyses of vaginal canal tissue samples collected from BALB/c mice in which vaginal candidiasis was induced but not treated showed an established infection by *Candida albicans*, with several yeasts and the formation of pseudohyphae/hyphae (**Figure 7A**). A clear reduction in the vaginal fungal burden was observed in the group of animals treated with the topical cream containing MCZ 2% (**Figure 7B**). Mice submitted to treatment with a topical cream containing 5% of C7a also showed a robust decrease in the vaginal fungal burden when compared to the untreated group (**Figure 7C**).

## Discussion

In the present study, we evaluated the antifungal activity of the biphosphinic cyclopalladated compound C7a against *Candida* spp. *in vitro* and *in vivo*. C7a was tested *in vitro* against 56 clinical and ATCC standard isolates of *C. albicans*, *C. tropicalis*, *C. parapsilosis*, *C. krusei*, and *C. glabrata* (susceptible- and resistant-azoles strains) and *in vivo* against the systemic and vaginal experimental infections induced by *C. albicans.*

We observed that C7a has an antifungal effect on different strains of *Candida* spp., even on those that are FCZ resistant. The MIC range of C7a on clinical isolates was of 0.25–4.0 μg/mL for *C. tropicalis*, *C. glabrata*, *C. krusei*, *C. parapsilosis*, and *C. albicans*, and similar MIC range was obtained with AMB, with no statistical difference between them (*p* = 0.056) (**Table 1**). MIC values obtained with MCZ and FCZ were broader, suggesting variability on the isolates’ response to these antifungals (**Table 1**). Although there was not a statistical difference between MIC values of C7a and MCZ (*p* = 0.9383), the difference between FCZ and C7a MIC values was significant, demonstrating that C7a was more efficacious, mainly against FCZ-resistant isolates. Previous work has also demonstrated the efficacy of C7a against *P. brasiliensis* and *P. lutzii* isolates *in vitro* at low concentrations (IC_50_ from 0.04 to 0.43 μg/mL and IC_90_ from 0.1 to 0.76 μg/mL) (Arruda et al., 2015).

The fungicidal activity of C7a and AMB, the most efficacious compounds against these isolates, was evaluated. On **Table 2**, we observed that the activity of both compounds on *C. tropicalis*, *C. glabrata*, and *C. krusei* was similar (*p* = 0.8399); however, while C7a was much more efficacious against clinical isolates of *C. albicans*, AMB showed stronger activity on *C. parapsilosis* clinical isolates.

The ability of *C. albicans* to infect diverse host niches is supported by a wide range of virulence factors and fitness attributes, as the morphological transition between yeast and filamentous forms, the expression of adhesins and invasins on the cell surface, the formation of biofilms, the secretion of hydrolytic enzymes, metabolic flexibility, amongst others (Mayer et al., 2013).

We evaluated the effect of C7a on the production of hyphae/pseudohyphae by *C. albicans*. Through calcofluor white staining, we observed that C7a decreased the *in vitro* formation of hyphae and pseudohyphae of *C. albicans* by 40% (**Figure 1E**). Microscopic analysis showed that the untreated control group presented an intense staining by calcofluor white on the hyphae tips, meaning that these regions are rich in chitin and are in growing phase (Gil et al., 1990). On the other hand, calcofluor white staining was present only in the mother yeast cell and not in the hyphae tips in the C7a-treated group. Moreover, yeast cells synthesize a chitin-rich septum during cell division (Cabib, 2004), and this structure was not observed in C7a-treated *C. albicans* (**Figure 1D**). This indicates that the compound inhibited the formation of hyphae and/or pseudohyphae, and even when these structures were formed they were not actively growing (**Figures 1A–D**).

Another important virulence factor of *Candida* species is biofilm formation which can confer resistance to some antifungal drugs such as FCZ (Mukherjee et al., 2003). To evaluate the effect of C7a on this virulence factor of *C. albicans*, we used a dual approach by employing two stages of biofilm development in order to correlate the effect of C7a on the initial and mature biofilm phases compared to AMB. We demonstrated that C7a inhibited the formation of the biofilm within 24 h at a BMIC of 1 μg/mL. The same concentration reduced the viability of *C. albicans* yeast cells already established in a mature biofilm (**Figure 3**). These results indicate that C7a is efficacious against *C. albicans* planktonic cells, inhibiting the biofilm formation, as well as against biofilm organisms, which present increased resistance to antifungals compared to their planktonic counterpart.

Cellular and molecular alterations have been shown after C7a-treatment of tumor cells and microorganisms. In C7a-treated *P. brasiliensis* yeast cells, the induction of chromatin condensation, DNA degradation, superoxide anion production and increased metacaspase activity were observed, as well as increased autophagic vacuole numbers and acidification, suggestive of apoptosis and autophagy, respectively (Arruda et al., 2015). Alterations like mitochondrial swelling, formation of abnormal membrane structures and atypical vacuoles were also observed in the trypomastigote form of *T. cruzi* parasites when treated with C7a (Matsuo et al., 2010). C7a-treated tumor cells showed similar alterations, as well as dissipation of the mitochondrial membrane potential, leading to Bax translocation, increased cytosolic calcium concentration, decreased ATP levels, activation of effector caspases, chromatin condensation and DNA degradation (Serrano et al., 2011).

TEM results showed that C7a induces morphological changes in *C. albicans* cells, similar to that observed previously in other microorganisms and tumor cells. Using 0.5 μg/mL of C7a, we observed a damage to the cell wall, mitochondrial apparent swelling, DNA alterations such as chromatin marginalization inside the nucleus, and a significant increase of electro-lucent vacuoles (**Figure 2** and **Supplementary Figure S1**).

Animals intravenously infected with *C. albicans* (ATCC 10231) yeast cells and intraperitoneally treated with C7a (0.3 mg/kg) showed a significant increase in survival compared to control animals (78% survival 30 days after infection vs. 0% survival 21 days after infection, respectively; **Figure 5**). C7a also significantly reduced the fungal burden in kidneys of systemically infected animals (**Figure 4**). When compared to FCZ, no statistical difference was observed for fungal burden and survival of the systemic candidiasis.

C7a was also efficacious in an experimental model of vaginal candidiasis and topical treatment with a vaginal cream, showing a significant decrease in colony forming units when compared with vehicle treatment (control group) (**Figure 6**). Histopathological analysis of infected tissue confirmed the therapeutic effect of C7a, leading to fungal elimination in the vaginal canal (**Figure 7**). When compared to MCZ, there was not statistical difference between this drug and C7a for treatment of vaginal candidiasis.

In addition, C7a has demonstrated low toxicity *in vivo*. Arruda et al. (2015) described that the compound was not toxic to animals when 600 μg/kg was administered i.p. sequentially for 5 days, with no morphological alterations in the kidney, liver, heart, lung, or spleen tissue. Moreover, Rodrigues et al. (2003) described a dose-escalating experiment, where mice showed no histological alterations in liver, lungs, kidney, heart, and spleen, after being inoculated three times per week with increasing doses of C7a, starting at 5 and ending at 60 μM.

In summary, the biphosphinic cyclopalladated C7a can be an alternative to treat candidiasis by *Candida* species, including *Candida* non-*albicans* and azoles-resistant isolates, mainly due to the low MIC values, fungicidal effect and low toxicity. The C7a mechanism of action on *Candida* spp. cell death remains to be completely determined, however, C7a is able to cause important morphological alterations, mainly inhibiting hyphae/pseudohyphae and biofilms. Thus, C7a can be a promising antifungal candidate for the treatment of fungal infections associated to the biofilms, such as systemic and vaginal candidiasis.

## Author Contributions

JM and DR designed and performed experiments, analyzed data and wrote the manuscript. KI and MM designed and supervised experiments, analyzed data. CS performed experiments, analyzed data. DG synthesized and purified compound C7a. AC designed and supervised synthesis of C7a. CT designed and supervised experiments, analyzed data. ER conceived the study, participated in its design and coordination, wrote the manuscript.

## Conflict of Interest Statement

The authors declare that the research was conducted in the absence of any commercial or financial relationships that could be construed as a potential conflict of interest.

## Supplementary Material

**FIGURE S1 | Additional images showing morphological alterations in C7a-treated cells.** Transmission electron microscopy of *Candida albicans* ATCC 10231 yeast cells untreated **(A–C)** and treated with 0.5 μg/ml of C7a **(D–F)** for 24 h at 37°C in RPMI 1640 buffered with MOPS 0.16 M. n, nucleus; m, mitochondria; v, vacuole. Arrowheads, damage at cell wall. Bars = 1 micrometers.
