# Supplementary figures and images for: Antifungal Activity of the Biphosphinic Cyclopalladate C7a against Candida albicans Yeast Forms In Vitro and In Vivo
Source: Front Microbiol. 2017 May 3;8:771. doi: 10.3389/fmicb.2017.00771 (PMC5413578; doi:10.3389/fmicb.2017.00771)

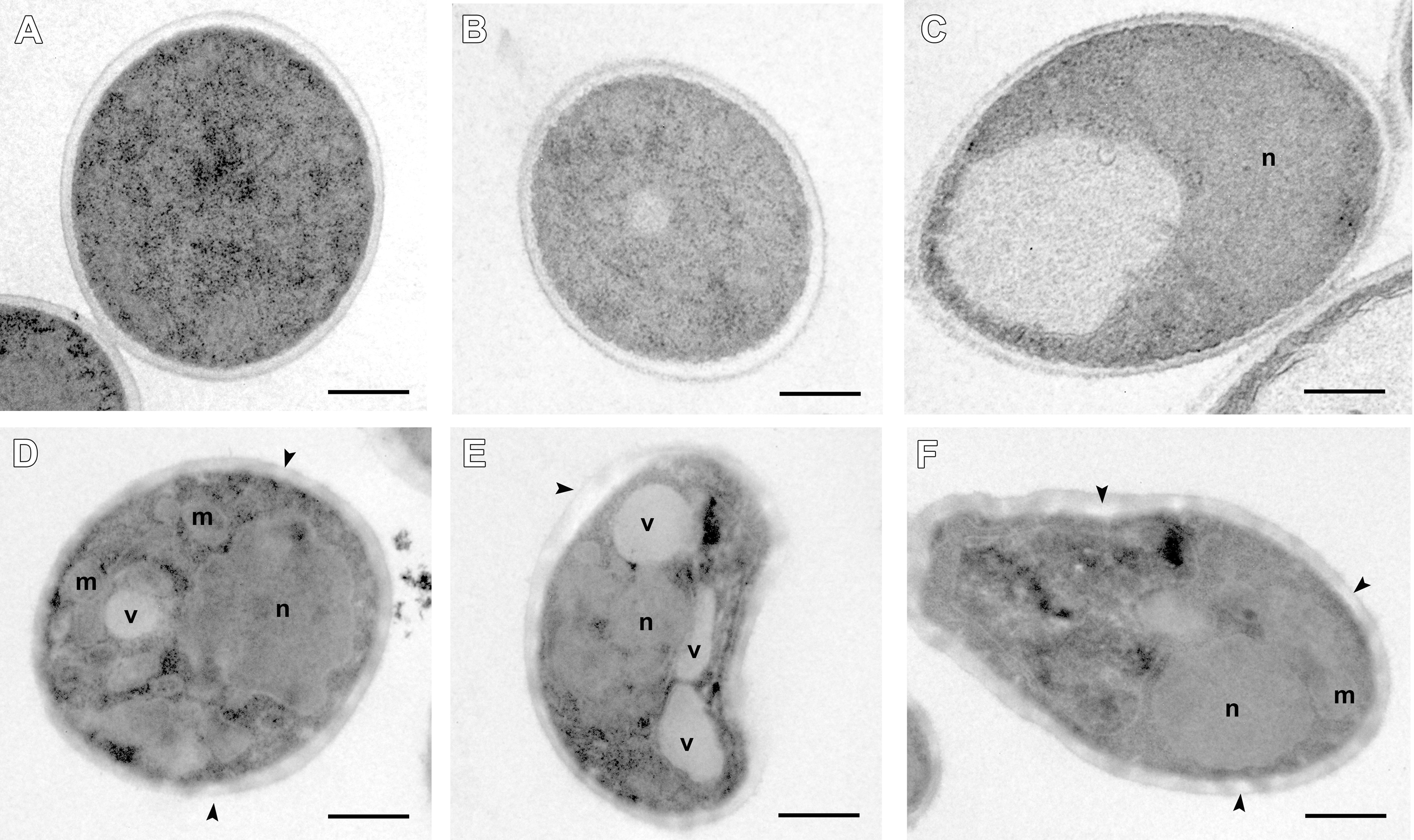

Supplement: Supplementary file 1 [file Image_1.TIF]
