# Supplementary material for: Antifungal Activity of the Biphosphinic Cyclopalladate C7a against Candida albicans Yeast Forms In Vitro and In Vivo
Source: Front Microbiol. 2017 May 3;8:771. doi: 10.3389/fmicb.2017.00771 (PMC5413578; doi:10.3389/fmicb.2017.00771)
Supplement: Supplementary file 2 [file Table_1.DOCX]

**Table S1.** MIC and MFC values for each compound and isolate, individually. ***R***, resistance; ***SDD,*** susceptible-dose-dependent, according document M27-S4 (CLSI, 2012).

|  | ***C7a*** | | ***AMB*** | | ***MCZ*** | | ***FCZ*** | |
| --- | --- | --- | --- | --- | --- | --- | --- | --- |
| **Strains** | **MIC** | **MFC** | **MIC** | **MFC** | **MIC** | **MFC** | **MIC** | **MFC** |
| ***C. albicans (n=17)*** |  |  |  |  |  |  |  |  |
| **SC 5314** | 0,5 | 0,5 | 1 | >16 | 0,031 | >16 | 0,25 | >64 |
| **ATCC 10231** | 1 | 1 | 0,5 | 1 | 4 | >16 | 0,5 | >64 |
| **ATCC 24433** | 1 | 1 | 1 | 2 | 0,5 | >16 | 0,5 | >64 |
| **IAL-40** | 0,5 | 2 | 1 | 1 | 2 | >16 | 64 ^R^ | >64 |
| **IAL-41** | 0,25 | 0,25 | 1 | 1 | 0,031 | >16 | 64 ^R^ | >64 |
| **IAL-42** | 0,5 | 0,5 | 0,5 | 0,5 | 0,125 | 1 | 64 ^R^ | >64 |
| **IAL-43** | 0,5 | 0,5 | 0,5 | 0,5 | 0,031 | >16 | 0,031 | >64 |
| **IAL-47** | 0,25 | 0,25 | 1 | 1 | 0,062 | 8 | 0,25 | 64 |
| **IAL-48** | 0,25 | 0,25 | 1 | 1 | 0,031 | >16 | 0,25 | >64 |
| **IAL-49** | 0,5 | 0,5 | 1 | 1 | 0,031 | >16 | 0,25 | >64 |
| **IAL-50** | 0,5 | 0,5 | 1 | 1 | 0,031 | >16 | 0,25 | >64 |
| **IAL-51** | 0,5 | 0,5 | 1 | 1 | 0,031 | >16 | 0,25 | >64 |
| **IAL-52** | 0,25 | 0,25 | 0,5 | >16 | 0,031 | 2 | 0,25 | >64 |
| **IAL-53** | 0,25 | 0,25 | 0,5 | >16 | 0,031 | >16 | 0,25 | >64 |
| **AL-54** | 0,5 | 0,5 | 0,5 | >16 | 0,031 | 8 | 0,5 | >64 |
| **IAL-55** | 0,25 | 0,25 | 0,5 | >16 | 0,031 | >16 | 0,125 | >64 |
| **IAL-56** | 0,25 | 0,25 | 0,5 | >16 | 0,031 | 4 | 0,125 | >64 |
| ***C. tropicalis (n=12)*** |  |  |  |  |  |  |  |  |
| **ATCC200956** | 0,5 | 2 | >16^R^ | >16 | >16 | >16 | 16 ^R^ | >64 |
| **ATCC28707** | 0,5 | 0,5 | 2^R^ | >16 | 4 | 8 | >64 ^R^ | >64 |
| **IAL-1** | 2 | 2 | 1 | 1 | 0,5 | 2 | 0,5 | >64 |
| **IAL-2** | 2 | 2 | 1 | 1 | 0,5 | 2 | 1 | >64 |
| **IAL-3** | 1 | 1 | 1 | 1 | 4 | >16 | 4 ^SDD^ | >64 |
| **IAL-4** | 1 | 1 | 1 | 1 | 8 | >16 | 64 ^R^ | >64 |
| **IAL-5** | 1 | 1 | 1 | 1 | 0,5 | >16 | 0,25 | 32 |
| **IAL-6** | 1 | >16 | 1 | 1 | 0,25 | 2 | 0,5 | >64 |
| **IAL-7** | 1 | 1 | 0,5 | 1 | 0,062 | 8 | 0,25 | >64 |
| **IAL-8** | 1 | 1 | 1 | 1 | 8 | >16 | 8 ^R^ | >64 |
| **IAL-9** | 1 | 1 | 1 | 1 | 0,25 | >16 | 1 | >64 |
| **IAL-10** | 0,5 | 0,5 | 1 | 1 | 0,062 | 4 | 0,25 | >64 |
| ***C.* *parapsilosis* (n=10)** |  |  |  |  |  |  |  |  |
| **ATCC22019** | 1 | >16 | 0,5 | 2 | 4 | >16 | 1 | 16 |
| **IAL-11** | 4 | 4 | 1 | 2 | 0,125 | 1 | 1 | 8 |
| **IAL-12** | 4 | 4 | 1 | 2 | 0,25 | 1 | 0,5 | 2 |
| **IAL-13** | 2 | 8 | 1 | 4 | 0,5 | 8 | 0,5 | 8 |
| **IAL-14** | 1 | 4 | 0,5 | 0,5 | 0,062 | 0,5 | 1 | 4 |
| **IAL-15** | 2 | 8 | 1 | 2 | 1 | 2 | 16 ^R^ | >64 |
| **IAL-16** | 4 | 4 | 1 | 2 | 0,062 | 0,25 | 0,062 | 0,5 |
| **IAL-17** | 4 | 16 | 1 | 1 | 4 | >16 | 64 ^R^ | >64 |
| **IAL-18** | 4 | 16 | 0,5 | 1 | 1 | >16 | 8 ^R^ | >64 |
| **IAL-19** | 1 | 1 | 1 | 1 | 0,062 | 2 | 0,5 | 36 |
| ***C.* *glabrata* (n=11)** |  |  |  |  |  |  |  |  |
| **ATCC 2001** | 2 | >16 | 0,125 | 4 | 16 | >16 | 8 | >64 |
| **IAL-20** | 0,5 | 0,5 | 1 | 1 | 0,06 | 8 | 2 | >64 |
| **IAL-21** | 0,25 | 0,5 | 2^R^ | 2 | 0,03 | 4 | 1 | >64 |
| **IAL-22** | 1 | 1 | 1 | 1 | 0,03 | 8 | 0,25 | >64 |
| **IAL-23** | 1 | 1 | 0,5 | 1 | 0,03 | 2 | >64 ^R^ | >64 |
| **IAL-24** | 1 | 1 | 0,5 | 1 | 0,03 | 8 | 1 | >64 |
| **IAL-25** | 1 | 1 | 1 | 1 | 0,06 | >16 | 2 | >64 |
| **IAL-26** | 4 | 4 | 1 | 2 | 0,5 | 2 | 0,25 | 2 |
| **IAL-27** | 1 | 1 | 0,5 | 1 | 0,06 | >16 | 4 | >64 |
| **IAL-28** | 1 | 1 | 0,5 | 1 | 0,03 | >16 | 0,25 | >64 |
| **IAL-29** | 0,5 | 1 | 0,5 | 0,5 | 0,03 | 8 | 2 | 64 |
| ***C.* *krusei* (n=11)** |  |  |  |  |  |  |  |  |
| **ATCC 6258** | 1 | >16 | 0,25 | 2 | >16 | >16 | 64 ^R^ | >64 |
| **IAL-30** | 1 | 2 | 0,5 | 0,5 | 2 | >16 | 64 ^R^ | >64 |
| **IAL-31** | 1 | 1 | 0,5 | 0,5 | 2 | >16 | >64 ^R^ | >64 |
| **IAL-32** | 1 | 1 | 0,5 | 0,5 | 4 | >16 | >64 ^R^ | >64 |
| **IAL-33** | 1 | 1 | 0,5 | 0,5 | 8 | >16 | >64 ^R^ | >64 |
| **IAL-34** | 1 | 1 | 0,5 | 1 | 2 | >16 | >64 ^R^ | >64 |
| **IAL-35** | 1 | 1 | 1 | 1 | 2 | >16 | 64 ^R^ | >64 |
| **IAL-36** | 1 | 1 | 1 | 2 | 2 | >16 | 64 ^R^ | >64 |
| **IAL-37** | 1 | 1 | 1 | 1 | 4 | >16 | 64 ^R^ | >64 |
| **IAL-38** | 1 | 1 | 1 | 1 | 4 | >16 | 64 ^R^ | >64 |
| **IAL-39** | 1 | 1 | 1 | 1 | 4 | 16 | 64 ^R^ | >64 |
